# Promoting nurse‐led behaviour change interventions to prevent cardiovascular disease in disadvantaged communities: A scoping review

**DOI:** 10.1111/hsc.13867

**Published:** 2022-06-13

**Authors:** Sarah Freeley, John Broughan, Geoff McCombe, Mary Casey, Patricia Fitzpatrick, Timothy Frawley, Janis Morrisey, J. T. Treanor, Tim Collins, Walter Cullen

**Affiliations:** ^1^ School of Medicine Health Sciences Centre, University College Dublin Dublin Ireland; ^2^ School of Nursing, Midwifery, and Health Systems Health Sciences Centre, University College Dublin Dublin Ireland; ^3^ School of Public Health, Physiotherapy and Sports Science, University College Dublin Health Sciences Centre Dublin Ireland; ^4^ St. Vincent's University Hospital Dublin Ireland; ^5^ Ireland East Hospital Group Dublin Ireland; ^6^ Irish Heart Foundation Dublin Ireland

**Keywords:** cardiovascular diseases, disadvantaged, health behaviour, practice patterns, nurses

## Abstract

Cardiovascular diseases (CVD) are the leading cause of death worldwide and they disproportionally affect people living in disadvantaged communities. Nurse‐led behaviour change interventions have shown great promise in preventing CVD. However, knowledge regarding the impact and nature of such interventions in disadvantaged communities is limited. This review aimed to address this knowledge gap. A six‐stage scoping review framework developed by Arksey and O'Malley, with revisions by Levac et al., was used. The search process was guided by the Preferred Reporting Items for Systematic Reviews and Meta‐Analyses‐Extension for Scoping Reviews (PRISMA‐ScR). Three electronic databases were searched (PUBMED/MEDLINE, CINAHL Plus, and Cochrane CENTRAL), and included studies were analysed using Braun and Clarke's ‘Thematic Analysis’ approach. Initial searches yielded 952 papers and 30 studies were included in the review following duplicate, title/abstract, and full‐text screening. The included studies indicate that nurse‐led behaviour change primary prevention interventions in disadvantaged areas are largely effective; albeit the considerable variety of intervention approaches, study populations and outcome measures used to date make it difficult to ascertain this. Other identified key areas in the promotion of nurse‐led behaviour change included tailoring interventions to specific populations, providing adequate training for nurses, overcoming patient access difficulties and encouraging patient engagement. Overall, the findings indicate that nurse‐led behaviour change interventions for high‐risk CVD patients in disadvantaged areas show much promise, although there is considerable variety in the interventions employed and studied to date. Further research is needed to examine the unique barriers and facilitators of interventions for specific disadvantaged groups.

**What is known about this topic?**

- Cardiovascular diseases' (CVD) impacts on mortality and quality of life are considerable, but nurse‐led behaviour change interventions to prevent CVD show promise. Still, understanding of nurse‐led behaviour change interventions for CVD patients in disadvantaged communities is limited.

**What this paper adds?**

- This study shows that primary prevention interventions have potential, although further research is needed to better understand the unique barriers and facilitators of these for specific disadvantaged groups.

## 1 INTRODUCTION

Cardiovascular diseases (CVD) are the primary cause of death globally and account for an estimated 17.9 million deaths each year (Mensah et al., 2019; Smyth et al., 2017). Two‐thirds of cardiovascular disease, deaths and cases are attributable to preventable risk factors (Jennings, 2014), and low socio‐economic status is an established and important risk factor for CVD development (Franks et al., 2011; Schultz et al., 2018). While policy guidelines for the management of high‐risk CVD patients have been proposed (Piepoli et al., 2016), they have not yet been properly established in clinical practice (Kotseva et al., 2019). Nonetheless, research indicates that nurse‐led primary prevention interventions show promise with regard to improving CVD patients' health outcomes. The World Health Organisation identifies greater utilisation of nurses, rather than physicians, as a potentially effective strategy for improving CVD care access in deprived areas (Schultz et al., 2018), and it has been claimed that nurses are ideally positioned to lead CVD prevention initiatives, as they are often the primary point of contact with patients and their families (Berra, 2011). The EUROACTION trial (2003–2006) meanwhile showed that a 16‐week nurse‐coordinated, multidisciplinary, family‐based programme could be more effective than usual care in preventing CVD (Wood et al., 2008), and a recent systematic review demonstrated that community‐based nurse‐led interventions are largely effective in reducing CVD risk (Tan et al., 2020). Tan et al.’s findings show that CVD risk can be reduced with respect to a number of outcomes including HbA1c reductions for diabetes patients, achievement of desired blood pressure goals for hypertensive patients and improvements around hyperlipidaemia patients' self‐reported dietary intake. Furthermore, Tan et al. indicate that effective prevention interventions apply a targeted approach towards underserved populations, and that appropriate funding, thoughtful design and training opportunities for nurses are needed to ensure optimal intervention outcomes.

However, knowledge regarding the impact and defining characteristics of nurse‐led behaviour change interventions for patients with high CVD risk in disadvantaged communities is lacking. This study aimed to address this problem and to inform clinical practice, policy and future research by examining the question; “How can nurse‐led behaviour change primary prevention initiatives enhance health outcomes among high CVD risk patients in disadvantaged communities?”

## 2 METHODS

A scoping review methodology using the six‐stage process developed by Arksey and O'Malley (Arksey & O'Malley, 2005), with revisions by Levac et al. (2010) was chosen to achieve an overview of existing literature on the topic of nurse‐led behaviour change interventions for high CVD risk patients in disadvantaged communities. Scoping reviews are well suited to the study of relatively poorly understood topics like this, particularly with regard to collating relevant literature in the area, and identifying key ideas and research gaps (Arksey & O'Malley, 2005).

### 2.1 Stage 1: Identifying the research question

This study's research question was formulated by identifying gaps in the existing literature and by consulting with healthcare professionals working in the areas of primary care, nursing, public health and health promotion. During these processes, consensus emerged that prevention of chronic diseases such as CVD among high‐risk groups is a population health priority, and that patients living in disadvantaged areas have a particularly high risk of developing CVD. It was also agreed that nurse‐led interventions show considerable promise with regard to tackling these issues. Thus, the following research question was formulated:

“How can nurse‐led behaviour change primary prevention initiatives enhance health outcomes among high CVD risk patients in disadvantaged communities?”

The following definitions were used:

*Nurse‐led* = Where nurses are the primary healthcare providers responsible for leading, coordinating, managing, delivering or facilitating patient care.

*Disadvantaged Community* = An area of low socio‐economic status. We chose to focus on income as an indicator of socio‐economic status, as some studies were unclear about how other factors (e.g. belonging to an ethnic minority group) could relate to socio‐economic status (Galobardes et al., 2007).

### 2.2 Stage 2: Identifying relevant studies

**FIGURE 1:**
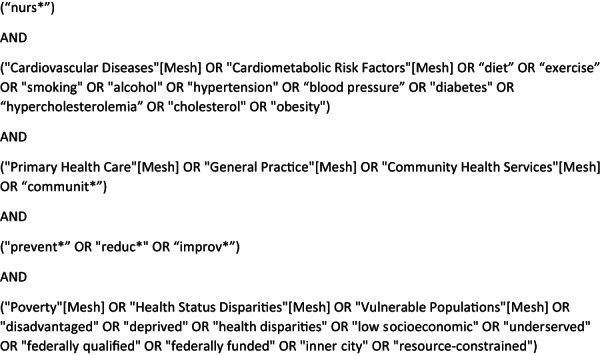
Search terms used

Initial searching of key databases was performed using multiple terms, and a short reading list was generated containing relevant studies. Search terms and MeSH terms were then generated by examining the studies' titles, abstracts and methodologies (See Figure 1). These search / MeSH terms were then used to conduct a second and more thorough literature search of electronic databases (i.e. “PUBMED/MEDLINE”, “CINAHL Plus” and “Cochrane CENTRAL”). Additional studies were added by searching grey literature using “Google” and by hand‐searching the reference sections of identified key literature.

### 2.3 Stage 3: Selecting studies

**FIGURE 2:**
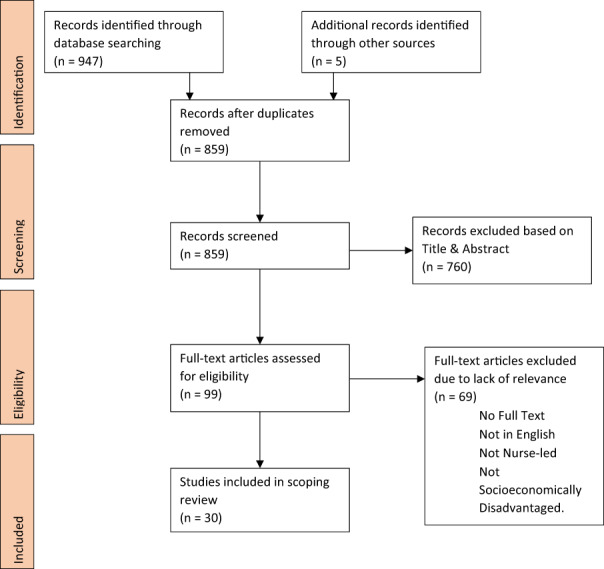
PRISMA ScR flow of identification, screening, eligibility, and inclusion

The study selection process consisted of a title / abstract review followed by a full‐text review. The selection pathway is summarised by the accompanying “PRISMA extension for Scoping Reviews” (PRISMA ScR) flow diagram (Figure 2). As is tradition with scoping reviews, both peer‐reviewed and grey literatures were included to facilitate incorporation of diverse research methodologies (Arksey & O'Malley, 2005), as were studies which aimed to reduce modifiable CVD risk factors including uncontrolled diabetes mellitus, hypertension, hypercholesterolemia, poor diet, lack of exercise, smoking and excessive alcohol consumption (Yusuf et al., 2004). Study protocols were not included. EndNote X9 software was used to assist in the screening process by tracking studies and managing citations.

The studies were selected according to predetermined inclusion and exclusion criteria which are outlined below:

### 2.4 Stage 4: Charting the data

The following data were extracted from included articles to facilitate analysis of included studies' findings: Author(s), year of publication; Study Title; Journal/Publication; Location; Study Design; Population; Type of Nurse; Recruitment Method; Intervention/Control; Main Results (see Table 2).

### 2.5 Stage 5: Collating, summarising and reporting results

Data from the included studies was collated, presented and analysed to provide an overview of the literature. From this, major themes in the literature regarding practice nurse‐led CVD primary prevention behaviour change interventions were identified using Braun and Clarke's “Thematic Analysis” approach (Braun & Clarke, 2006).

### 2.6 Stage 6: Consultation

As per the guidance of Levac et al. (Levac et al., 2010), consultation with experts in the areas of primary care, nursing, public health and health promotion was conducted, and studies were included/excluded and interpreted based on their advice.

## 3 RESULTS

### 3.1 Search results

Initial searching of “PUBMED/MEDLINE”, “CINAHL Plus” and “Cochrane CENTRAL” yielded 947 results, while hand‐searching of grey literature and the references of key literature identified five more studies. Duplicates were then removed (*n* = 93), and the titles / abstracts of 859 papers were screened for relevance. Seven hundred and sixty of these were excluded due to not meeting study inclusion criteria (see Table 1). The remaining 99 studies then underwent full‐text review by two independent reviewers (SF & JB), and 69 were removed due to not meeting study inclusion criteria. Thirty studies were included for final analysis (see Figure 2).

**TABLE 1:** Study inclusion and exclusion criteria

Inclusion criteria	Exclusion criteria
Published in English	Not available in English
Full text available	Full text not available
Published between 2006–2021	Published before 2006
Examined nurse‐led interventions	Did not examine nurse‐led interventions
Focused on adults	Focused on children
Examined behaviour change that prevents CVD/ reduces risk factors for CVD	Did not examine behaviour change that prevents CVD / reduces risk factors for CVD
Conducted in a community healthcare setting	Conducted in a hospital setting
Focus on primary prevention of CVD	Did not focus on primary prevention of CVD
Conducted in a socio‐economically disadvantaged community	Not conducted in a socio‐economically disadvantaged community

### 3.2 Description of included studies

Various research designs were used and are broadly categorised as the following: randomised control trials (*n* = 12); undefined intervention study (*n* = 4); pre‐post design (*n* = 6); qualitative study (*n* = 2); cohort study (*n* = 4); pilot study (*n* = 1); and cross‐sectional survey (*n* = 1). The studies examined reduction of overall CVD risk (*n* = 10), improved diabetes control (*n* = 10), hypertension reduction (*n* = 5), smoking cessation (*n* = 4) and reduced alcohol consumption (*n* = 3). Twenty‐seven studies examined the effectiveness of nurse‐led interventions, one study focused exclusively on services delivered by nurses (Monay et al., 2010) and two studies investigated nurse‐led care barriers (Derksen et al., 2019; Jansen et al., 2007). Nurses involved in the studies were practice nurses (*n* = 4), nurse practitioners (*n* = 5), public health or community health nurses (*n* = 3), visiting nurses (*n* = 3), other specialised nurses (*n* = 4), faith community nurses (*n* = 1), telehealth nurses (*n* = 1), nurse case managers or nurse champions (*n* = 5) and “other” nurses (*n* = 6). Studies were conducted in the USA (*n* = 16), the UK (*n* = 4), South Korea (*n* = 4), Ireland (*n* = 1), South Africa (*n* = 1), the Netherlands (*n* = 3) and Sweden (*n* = 1) (see Table 2).

**TABLE 2:** Description of included studies

Author/Year	Study title	Journal/Publication	Location	Study design	Population	Type of nurse	Method of recruitment	Intervention/Control	Main results
Allen et al. (2011)	*Community outreach and cardiovascular health (COACH) trial: a randomised, controlled trial of nurse practitioner/community health worker cardiovascular disease risk reduction in urban community health centres*.	Circulation: Cardiovascular quality and outcomes	Baltimore, USA	Randomised, controlled trial	Patients attending federally funded community health clinics. (*n* = 525)	Nurse practitioner	Patients were recruited from two community health centres and were identified from clinic‐based computerised ICD 9 codes	The participants were stratified by race and sex, then randomly assigned to receive the nurse practitioner/ community healthcare worker intervention or enhanced unusual care (control)	The intervention used can be effective in reducing CVD risk factors and improving perceptions of chronic illness care in high‐risk patients.
Bachhuber et al. (2017)	*Delivery of screening and brief intervention for unhealthy alcohol use in an urban academic federally qualified health centre*.	Addiction science & clinical practice	New York, USA	Intervention study	Adult patients who attended a federally qualified health centre in the Bronx, NY between October 2013 and September 2014.(*n* = 9119)	Nurse Champion	Patients who attended the federally qualifies health centre were recruited.		The nurse‐delivered screening and brief intervention model led to nearly half of eligible patients being screened and over half of those who screened positive being provided with a brief intervention.
(Beckham, 2007)	*Motivational interviewing with hazardous drinkers*	Journal of the American Academy of Nurse Practitioners	Idaho, USA	Randomised control trial	Hazardous drinkers attending five low‐income community health centres.(*n* = 26)	Family Nurse Practitioners	The Alcohol Use Disorders Identification Test (AUDIT) was used to screen participants' alcohol use. If a participant had an AUDIT score which indicated that they were a hazardous drinker then they were recruited into the study.	The intervention group had one motivational interviewing session with the nurse practitioner.The control group had no treatment.	The intervention was successful in improving the drinking pattens of hazardous drinkers in this study.
Brown et al. (2011)	*Integrating education, group support, and case management for diabetic Hispanics*.	Ethn Dis	Texas‐Mexico border, USA	Pre‐post control group design	Mexican American adults living in a low‐income, rural community on the Texas‐Mexico border(*n* = 165) (83 participants and 82 supporters)	Nurse Case Manager	Participants were recruited from rosters of ongoing genetic and epidemiological studies conducted by one of the authors of the study.	Both groups received a Culturally tailored diabetes self‐management education intervention. The intervention group additionally had access to a nurse case manager while the control group did not.	Higher levels of contact with the nurse case manager resulted in increased attendance at the culturally tailored education intervention. Greater attendance resulted in improved health outcomes.
Carter et al. (2011)	*A patient‐centric, provider‐assisted diabetes telehealth self‐management intervention for urban minorities*.	Perspect Health Inf Manag	Washington DC, USA	Randomised controlled trial	African American patients with type 2 diabetes living in medically underserved low‐income communities in the inner‐city (*n* = 47)	Telehealth nurse	Diabetic patients were recruited from one primary care practice in Washington, DC and were then assessed for eligibility.	The intervention group was provided with equipment for monitoring their health data and uploading it to their patient record. They met with the telehealth nurse and used a portal containing a self‐management module, a health education module and a social networking module. The control group received standard care from their providers but had no access to the online portal and no interaction with the telehealth nurse.	The intervention group was more likely to achieve positive health outcomes (lowered haemoglobin A1c and BMI) than were control group members.
(Choi & Rush, 2012)	*Effect of a short‐duration, culturally tailored, community‐based diabetes self‐management intervention for Korean immigrants: a pilot study*.	The Diabetes Educator	USA	Pre‐post design	Korean immigrant adults (predominantly low‐income) from an underserved Korean community on the West Coast (*n* = 53)	Nurse Practitioners	The sample was recruited using flyers, centre newsletters, and Korean newspapers.	The intervention was two diabetes self‐management sessions which were led by an experienced bilingual family nurse practitioner. A follow‐up session was conducted three months after the intervention to assess the long‐term effects.	The intervention was effective in improving physiological outcomes and self‐care behaviours.
Connolly et al. (2017)	*Outcomes of an integrated community‐based nurse‐led cardiovascular disease prevention programme*.	Heart	Westminster, UK	Intervention study	Patients from three community hubs in a deprived district (Westminster). (*n* = 3232)	Cardiovascular Nurse Lead Nurse	The MyAction programme was conducted in three community hubs. Eligible patients were referred from primary and secondary care. They were contacted in person and in writing by the referral coordinator.	There was no control group. The intervention consisted of a 12‐ week programme which included individual follow‐up sessions, an educational workshop each week and supervised exercise sessions using simple equipment.	The nurse‐led, multidisciplinary approach resulted in favourable changes in lifestyle and medical risk factors, the prescription of cardioprotective medication and improvements in patient‐reported outcome measures.
Crowley et al. (2013)	*The Cholesterol, Hypertension, And Glucose Education (CHANGE) study: results from a randomised controlled trial in African Americans with diabetes*.	American Heart Journal	North Carolina, USA	Randomised Controlled Trial	African American patients with Type 2 Diabetes Mellitus receiving care at a primary care clinic (*n* = 359)	Nurses	Eligible patients were sent a letter requesting study participation from investigators and their primary care physician.	The intervention group received monthly self‐management education through a telephone call from the nurse and quarterly medication management facilitation via electronic communication with the nurse ad primary care physician. The control group received usual care.	The intervention improved self‐reported medication adherence but not CVD risk factor control.
Dean et al. (2014)	*Evaluation of a specialist nurse‐led hypertension clinic with consultant backup in two inner city general practices: randomised controlled trial*.	Family Practice	UK	Randomised Controlled Trial	Patients with hypertension were recruited from two multi‐ethnic, inner London general practices. (*n* = 353)	Specialist Nurse	Eligible patients were randomly selected by the hypertension specialist nurse and assigned to the intervention or control group. Patients randomised to the intervention were sent an invitation letter by their GP.	Patients in the control group continued with usual GP care. Patients in the intervention group attended a weekly specialist nurse‐led hypertension clinic. Follow‐up by the nurse included motivational interviews and encouragement of behavioural change to reduce CVD risk factors.	The specialist nurse‐led hypertension clinic was associated with reduced blood pressure in patients compared with usual care. Almost half of the patients randomised to the intervention did not attend but it is noted that patients may have been more likely to attend a clinic run by a practice nurse with whom they were familiar.
Derksen et al. (2019)	*Barriers experienced by nurses providing smoking cessation support to disadvantaged, young women during and after pregnancy*.	Health & social care in the community	Netherlands	Qualitative observational study	Nurses working within a Dutch preventive care programme for disadvantaged young women (VoorZorg)(*n* = 16)	Certified specialised nurses	Managers of youth care organisations executing the VoorZorg programme were informed about the study and asked for permission of their nurses to participate in this study.	N.A.	The smoking cessation programme could be improved by improving training for nurses.
El Fakiri et al. (2008)	*Intensified preventive care to reduce cardiovascular risk in healthcare centres located in deprived neighbourhoods: a randomised controlled trial*.	European Journal of Cardiovascular Prevention & Rehabilitation	Netherlands	Randomised Controlled Trial	Patients living in deprived neighbourhoods 30–70 years of age, with registered cardiovascular risk factors or diseases. (*n* = 275)	Practice nurse	GPs' databases were searched to identify patients who met the eligibility criteria. Patients were assigned to usual care or intervention and research assistants approached the patients in the intervention group to obtain signed informed consent.	The control group received usual care. The intervention was delivered by a Practice Nurse‐led team who followed a structured protocol which included a ‘treatment plan’ tailored to the patient's risk factors.	There was no statistically significant difference between the absolute risk of CVD in the control group and intervention group following treatment. Both groups had significant reductions in CVD risk. The lack of a difference between groups may be explained by an inadequate delivery of the intervention.
Fischer et al. (2012)	*Nurse‐run, telephone‐based outreach to improve lipids in people with diabetes*.	Am J Manag Care	Denver, USA	Prospective randomised controlled trial	Patients with type I or II diabetes and predominantly had low‐income and were Latino. (*n* = 762)	Registered nurses trained in algorithms for diabetes care.	Patients were identified through the Westside Clinic Diabetes Registry and assessed for eligibility.	The intervention was a telephone outreach programme used in addition to usual care. This outreach programme involved motivational interviewing to help patients achieve targets. The control group received usual care.	Nurses can improve lipid control in patients with diabetes in a low‐income population through telephone care and the use of algorithms. A more targeted approach is needed to achieve better outcomes.
Froelicher et al. (2010)	*Combining community participatory research with a randomised clinical trial: the Protecting the Hood Against Tobacco (PHAT) smoking cessation study*.	Heart Lung	San Francisco, California, USA	Randomised Clinical Trial	Patients who use tobacco and live in a low‐income community (*n* = 87)	Community Health Nurse	Patients were recruited using flyers, a website and public service announcements on local public access television and radio stations.	The control group was allocated to a smoking cessation programme. The intervention group received this same programme but supplemented by a tailored, community co‐developed tobacco industry and media related smoking cessation intervention.	The intervention group had a higher smoking cessation rate than the control group. The recruitment strategies were successful in recruiting patients from an underserved, inner‐city community.
Gary et al. (2009)	*The effects of a nurse case manager and a community health worker team on diabetic control, emergency department visits, and hospitalizations among urban African Americans with type 2 diabetes mellitus: a randomised controlled trial*.	Archives of internal medicine	Maryland, USA	Randomised Controlled Trial	African American patients with type 2 Diabetes Mellitus attending primary care clinics in medically underserved areas (*n* = 542)	Nurse Case Manager	Patients were identified through administrative databases and screened for eligibility by telephone.	The control group received minimal care. This consisted of mailings and telephone calls every 6 months to remind them about screenings. The intervention group received minimal care plus individualised, culturally tailored care provided by a nurse case manager and community health worker.	The intervention reduced Emergency Department visits. Greater contact with the nurse case manager and community health worker seemed to have the greatest benefit in improving HbA1c levels.
(Gibson, 2008)	*Heart Smart. A two‐year report on a community‐based cardiovascular disease prevention programme in the West of Ireland*.	Croí West of Ireland Cardiology Foundation	Galway, Ireland	Cohort study	A representation of urban and rural areas in Galway, Mayo, Roscommon, Clare and Donegal. A special emphasis being placed on those from the lower socio‐economic groups. (*n* = 1491)	Primary Care Nurses	In the majority of cases, individuals opportunistically participated in the programme on a first‐come, first‐served basis.	The intervention group followed the nurse‐led Heart Smart Programme. This programme includes 3 components: identification of high risk, asymptomatic individuals, a 20‐minute risk factor assessment and lifestyle support using motivational interviewing techniques and a follow‐up at 6 months	After 2 years, the intervention had a significant impact in reducing the cardiovascular risk of participants.
Jansen et al. (2007)	*Tailoring intervention procedures to routine primary health care practice: an ethnographic process evaluation*.	BMC Health Services Research	Rotterdam and The Hague, Netherlands	Qualitative study	Researchers (*n* = 7) and practice nurses (*n* = 7) who had taken part in the Quattro Trial in deprived neighbourhoods.	Practice Nurses		N.A.	The implementation of the Quattro Project in deprived neighbourhoods was hindered by the lack of standard protocols.
Jeong et al. (2018)	*Telephone support and telemonitoring for low‐income older adults*.	Research in Gerontological Nursing	South Korea	A randomised, controlled, single‐blinded, parallel pilot trial	Low‐income older adults with Hypertension and Diabetes (*n* = 40)	Nurse	Patients were selected from a database of a national home telemonitoring project.	The control and intervention groups both had weekly health education. The intervention group additionally received a 30‐minute telephone call from a nurse twice a week for eight weeks. During these calls, the nurse helped the patient to establish goals, communicate barriers and monitor risk factors. The nurse educated the patient with regard to self‐management, encouraged health maintenance and rewarded goal attainment.	Nurse‐led telephone support may be effective in improving health behaviour, systolic blood pressure, and hypertension self‐care in disadvantaged older adults.
Jordan et al. (2011)	*An evaluation of Birmingham Own Health® telephone care management service among patients with poorly controlled diabetes. a retrospective comparison with the General Practice Research Database*.	BMC Public Health	UK	Retrospective cohort study	Patients aged ≥18 years with diabetes enrolled onto Birmingham Own Health® (*n* = 473) were each matched to up to 50 patients with diabetes registered with the General Practice Research Database (*n* = 21,052). Birmingham Own Health® was directed towards practices in the most deprived areas.	Nurse Care Manager	Patients in the Birmingham Own Health® diabetes module were referred to the programme by their GP.	The intervention group had taken part in the Birmingham Own Health® diabetes module so had received self‐management support motivational telephone counselling from nurse care managers. The control group had not undertaken this programme.	The intervention was effective in reducing HbA1c levels, blood pressure and BMI in people with diabetes. The intervention showed to be effective in the most deprived populations.
Kim et al. (2014)	*Effects of community‐based case management by visiting nurses for low‐income patients with hypertension in South Korea*.	Jpn J Nurs Sci	Seoul, South Korea	Single group pre–post study	Newly registered low‐income adult patients with hypertension in a public health centre in Seoul (*n* = 22)	Visiting Nurse	Patients were selected from a database at the public health centre and were assessed for eligibility.	There was no control group. The intervention was a case management programme delivered by visiting nurses for 2–8 months which targeted blood pressure reduction, lifestyle modification, and improvement of knowledge on hypertension.	The intervention was effective in reducing blood pressure levels and increasing knowledge and self‐management level of the low‐income adults with hypertension in the community.
Ko et al. (2011)	*Effects of visiting nurses' individually tailored education for low‐income adult diabetic patients in Korea*.	Public Health Nurs	Korea	One‐group pre‐post study	Newly registered low‐income adult diabetic patients in a public health centre (*n* = 96)	Visiting Nurses	Eligible patients attending the public health centre were asked to participate.	Each visiting nurse delivered 7 monthly educational sessions to the same six‐seven patients over 8 months. The programme was individual tailored by the nurses after they assessed each participant's educational background and level of understanding, and family and environmental factors.	The tailored education programme had a positive impact on patients' knowledge of diabetes and self‐management.
McKee et al. (2011)	*A collaborative approach to control hypertension in diabetes: outcomes of a pilot intervention*.	J Prim Care Community Health	Bronx, New York, USA	Pilot intervention	Adults receiving care for type 2 diabetes at a federally qualified health centre. (*n* = 55) The sample was primarily low‐income and ethnically diverse.	Home health nurses and a nurse coordinator		In the intervention, home health nurses assessed patients' self‐management and adherence to medication. They also provided behavioural counselling. Patients transmitted health data to the nurse coordinator who transmitted these results weekly to the primary care provider. The control group received usual care.	The intervention proved to be effective in reducing blood pressure and improving blood glucose in this low‐income setting.
McRobbie et al. (2016)	*Tackling obesity in areas of high social deprivation: clinical effectiveness and cost‐effectiveness of a task‐based weight management group programme‐a randomised controlled trial and economic evaluation*.	Health technology assessment	London, UK	Randomised Controlled / Cost‐effectiveness analysis	Patients aged ≥ 18 years, wanted to lose weight and had a BMI of ≥ 30 kg/m2 or ≥ 28 kg/m2 plus comorbidities attending GP Practices in Tower Hamlets and Hackney, two deprived boroughs. (*n* = 330)	Practice Nurses	Posters/flyers in reception area and consultation rooms, adverts on GP practice website and boards, text and letter mailshots to potential participants identified via GP database searches, GP fax/telephone referrals, ‘comments’ box on GP reception allowing potential participants to express their interest.	The control group received ‘best practice’ weight management advice from Practice Nurses. The intervention group received the Weight Action Programme.	The Weight Action Programme intervention was more effective and more cost effective than weight loss advice delivered by a practice nurse.
Mertens et al. (2014)	*Effectiveness of nurse‐practitioner‐delivered brief motivational intervention for young adult alcohol and drug use in primary care in South Africa: a randomised clinical trial*.	Alcohol and alcoholism (Oxford, Oxfordshire)	Delft, South Africa	Randomised Clinical Trial	Patients ages 18–24 who visited a large public‐sector primary health care clinic (serving a low‐income population) and screened positive for alcohol or drug misuse. (*n* = 403)	Nurse Practitioner	Patients were screened for eligibility using an adaption of the single item alcohol screening question and an adaption of the single question drug screener.	The intervention group received a brief motivation intervention delivered by a nurse‐practitioner and a referral list for resources to combat drinking and drug use. The control group received minimally enhance usual care and a resource list.	The motivational interviewing may have been effective in reducing short‐term alcohol misuse by low‐income young adults in primary care. Evidence was not found for a reduction in drug misuse.
Monay et al. (2010)	*Services delivered by faith‐community nurses to individuals with elevated blood pressure*.	Public Health Nurs	Los Angeles, USA	Cross‐sectional survey	Patients with elevated blood pressure (mostly low‐income) who received a visit from faith community nurses (*n* = 33)	Faith Community Nurses	Patients were recruited from 26 health fairs at 11 churches in Los Angeles County.	N.A.	The services most frequently provided by these nurses were blood pressure measurement, hypertension‐specific education on dietary changes and supportive counselling.
(Murphy et al. (2015)	*Improving cardiovascular health of underserved populations in the community with Life's Simple 7*.	Journal of the American Association of Nurse Practitioners	USA	Cohort Study	Adults living in two inner city community sites (a senior centre servicing African American older adults, and a residential facility servicing homeless women) (*n* = 28)	Nurse Practitioners	Patients were identified by a health screening in the urban senior centre or the residential facility. Suitable patients were invited to participate.	Participants completed the Nurse Practitioner led American Heart Association 6‐month Healthy Heart Programme using the My Life Check and Life's Simple 7 tools.	African American older adults in the study experienced improvements in CVD risk factors. Women in the study did not experience these improvements.
Piñeiro et al. (2020)	*Implementation of Ask‐Advise‐Connect in a safety net healthcare system: quitline treatment engagement and smoking cessation outcomes*	Transl Behav Med	USA	Implementation trial	Adults attending 13 community clinics that provide care to low‐income, predominantly racial/ethnic minority smokers. (*n* = 4806)	Licensed vocational nurses	Patients who visited the clinic could enrol in the treatment.	Ask‐Advise‐Connect was implemented in 13 community clinics for a 34‐month period. The programme involved training medical staff to ask about smoking status, advise smokers to quit, and offer to immediately connect smokers with quit lines through an automated link within the electronic health record.	Treatment engagement was high compared to more traditional, referral‐based approaches in the literature. Self‐reported abstinence was in line with other studies but biochemically confirmed abstinence was much lower. Therefore, self ‐reporting may not be accurate in such studies.
Shishani et al. (2019)	*Quit Happens: A community clinic‐based, multitiered smoking cessation intervention*.	Public Health Nurs	Washington and Idaho, USA	Pre–post design	Adults who self‐reported being tobacco users and attended a federally qualified health centre with a low‐income population (*n* =	Specialised Public Health Nurse		The programme used the 5A model: “Asking patients whether they use tobacco”, “Advising patients to quit using tobacco”, “Assessing patients' readiness to quit”, “Assisting patients with quitting”, and “Arranging follow‐up”. Patients who were ready to quit were provided with an individualised quit plan and nicotine replacement therapy. Those who were not ready to quit were provided with written educational materials and a Quitline card.	The programme was successful in increasing smoking cessation among patients. A public health nurse can successfully provide training and programme development in a leadership role for such a programme.
Waller et al. (2016)	*A primary care lifestyle programme suitable for socio‐economically vulnerable groups ‐ an observational study*.	Scand J Prim Health Care	Gothernburg, Sweden	Cohort study	Adults (with different levels of socio‐economic vulnerability) who attended a primary health centre over an eight‐month period (*n* = 3691)	Nurse acting as a health educator		There was no control group. The intervention was a short health questionnaire and health dialogue with a nurse. Patients were classified according to four socio‐economic vulnerability factors: education, employment, ethnicity and living situation.	Lifestyle changes were achieved to the same extent in both higher and lower socio‐economic groups. The intervention was successful in reaching socio‐economically vulnerable patients.
(Weiler & Tirrell, 2007)	*Office nurse educators: improving diabetes self‐management for the Latino population in the clinic setting*	Hispanic Health Care International	Idaho, USA	Intervention study	Low‐income Latino patients with type 2 diabetes attending a rural community health centre (*n* = 20)	Office Nurse Educator (Licensed Practical Nurses and Registered Nurses)	Eligible patients were identified by retrospective chart review and computerised billing data from the clinic.	Bilingual Latina office nurses were trained as Certified Diabetes Educators and saw patients within the clinic at the time of regularly scheduled clinician appointments, during separately scheduled diabetes education appointments, or as part of a multidisciplinary Diabetes Emphasis Clinic.	Training office nursing staff as diabetes nurse educators in a low‐cost solution to successfully educating patients at a busy primary practice in management of their diabetes.
Yang et al. (2016)	*Effects of a South Korean community‐based cardiovascular disease prevention program for low‐income elderly with hypertension*.	Journal of Community Health Nursing	South Korea	A quasi‐experimental, between group, pre‐post design	Low‐income elderly patients that were registered for chronic disease management at a public health centre in the suburbs of Seoul and met the inclusion criteria and agreed to participate. (*n* = 91)	Community health nurses	Five trained visiting nurses recruited patients using a one‐to‐one interview method	The intervention was a 3‐month self‐efficacy enhancing programme which included 40‐minute personal visits by trained nurses and a 10‐minute follow‐up telephone call. The control group received standard care.	A CVD prevention programme implemented by a visiting nurse may be effective in improving self‐efficacy, health behaviour an CVD risk factors in a community setting.

### 3.3 Patient populations

#### 3.3.1 Person‐centred care

Many prevention interventions were tailored towards individual patient needs (Allen et al., 2011; Brown et al., 2011; Connolly et al., 2017; Crowley et al., 2013; El Fakiri et al., 2008; Gary et al., 2009; Jansen et al., 2007; Jeong et al., 2018; Jordan et al., 2011; Kim et al., 2014; Ko et al., 2011; Murphy et al., 2015). These interventions involved developing individualised care plans (Allen et al., 2011; El Fakiri et al., 2008; Jordan et al., 2011; Kim et al., 2014; Murphy et al., 2015), educational resources, follow‐up sessions (Connolly et al., 2017; Crowley et al., 2013; Gary et al., 2009; Jeong et al., 2018; Jordan et al., 2011; Ko et al., 2011; Murphy et al., 2015) and opportunities for patients to ask for individualised health guidance (Brown et al., 2011; Jordan et al., 2011).

#### 3.3.2 Older patients

Prevention interventions were also designed to meet the needs of specific patient groups. For instance, some studies targeted care for older populations (Jeong et al., 2018; Kim et al., 2014; Ko et al., 2011;Murphy et al., 2015; Yang et al., 2016). It was recognised that older patients are particularly vulnerable, as they often live alone (Jeong et al., 2018; Kim et al., 2014; Yang et al., 2016), suffer more frequently from cognitive and mood problems (Jeong et al., 2018), are more likely to have poor cardiovascular health (Murphy et al., 2015; Yang et al., 2016) and may have poorer health literacy and digital literacy (Jeong et al., 2018). Telephone supports were often used to combat these issues and were shown to be cost‐effective and useful (Jeong et al., 2018;Kim et al., 2014; Ko et al., 2011; Yang et al., 2016). The success of telephone supports was largely attributed to the fact that older adults are familiar with the technology, and because frequent contact of this nature can help develop trust in patient–care provider relationships (Jeong et al., 2018; Kim et al., 2014). Visiting nurses were also employed to combat travel‐related care access difficulties among this population (Kim et al., 2014; Ko et al., 2011; Yang et al., 2016), longer sessions were used to afford older people more time when learning as part of educational initiatives (Ko et al., 2011) and older patients were shown to enjoy using logs to track progress towards and achievement of goals (Murphy et al., 2015).

#### 3.3.3 Ethnic minorities

Seventeen studies focused on ethnic minority or ethnically diverse populations. These prevention interventions were often tailored for ethnic and cultural differences, values, beliefs and practices (Connolly et al., 2017; Crowley et al., 2013; Gary et al., 2009; Weiler & Tirrell, 2007). For instance, studies featured community healthcare workers (CHWs) with the same ethnicity as patients (Allen et al., 2011; Brown et al., 2011; El Fakiri et al., 2008; Gary et al., 2009; Jansen et al., 2007), interventions using patients' native language(s) (Choi & Rush, 2012; Piñeiro et al., 2020) and bilingual nurses (Bachhuber et al., 2017; Brown et al., 2011; Choi & Rush, 2012; Weiler & Tirrell, 2007). Traditional beliefs in relation to medical practice were also acknowledged (Choi & Rush, 2012), and culturally appropriate foods were recommended (Brown et al., 2011; Choi & Rush, 2012).

#### 3.3.4 Patients with Low‐Literacy/education

Five studies noted challenges when working with populations that have low health literacy and/or low educational status (Allen et al., 2011; El Fakiri et al., 2008; Jeong et al., 2018; Mertens et al., 2014; Murphy et al., 2015). Included studies suggest that patients with lower educational levels are less likely to participate in health education programmes (El Fakiri et al., 2008; Jeong et al., 2018) and prevention interventions were designed to counter this issue using accessible resources and strategies including low literacy guides (Allen et al., 2011; Crowley et al., 2013; Murphy et al., 2015) and interview rather than written questionnaire data collection methods (Mertens et al., 2014).

#### 3.3.5 Gender

The importance of making interventions attractive to both genders was not considered in most studies. However, one study noted that this issue should be addressed in future research, as more women are recruited for weight management programmes (McRobbie et al., 2016). Another study found that motivational interviewing interventions may be more effective in reducing alcohol and drug use among men rather than among women (Mertens et al., 2014), and one study involved implementing an intervention for pregnant women (Derksen et al., 2019).

#### 3.3.6 Rural patients

Four studies were conducted in rural settings (Beckham, 2007; Brown et al., 2011; Shishani et al., 2019; Weiler & Tirrell, 2007) and two others were not, but they did include participants from rural areas (Gibson, 2008; Waller et al., 2016).

### 3.4 Effectiveness

Twenty‐seven studies focused on assessing prevention interventions' effectiveness. Various effectiveness indices were used including clinical (*n* = 21), behaviour change (*n* = 17), attendance / engagement (*n* = 4) (Dean et al., 2014; Gibson, 2008; Piñeiro et al., 2020; Weiler & Tirrell, 2007), health knowledge (*n* = 4) (Carter et al., 2011; Choi & Rush, 2012;Kim et al., 2014; Ko et al., 2011), mood / emotional (*n* = 2) (Choi & Rush, 2012; Connolly et al., 2017) and hospital admission (*n* = 2) (Fischer et al., 2012; Gary et al., 2009) outcomes. Most studies determined that nurse‐led care was effective in improving all or some of the measured outcomes (*n* = 26). Factors which reportedly limited effectiveness included small sample size (Froelicher et al., 2010), interventions not being time‐intensive enough (Brown et al., 2011; Crowley et al., 2013; Fischer et al., 2012), patients having low education levels (El Fakiri et al., 2008), interventions not being delivered as planned, (El Fakiri et al., 2008; Jansen et al., 2007) and interventions not being tailored to specific populations (Murphy et al., 2015).

### 3.5 Cost‐effectiveness

Cost‐effectiveness was rarely examined by the included studies. However, one nurse practitioner and community health worker (CHW)‐led intervention was found to be a cost‐effective strategy for reducing cardiovascular risk in minority, underserved populations (Allen et al., 2011; Allen et al., 2014), whereas telephone supports (Brown et al., 2011; Jeong et al., 2018), short‐term interventions (Beckham, 2007) and visiting nurses (Kim et al., 2014; Ko et al., 2011) were all reported as low‐cost initiatives. Two studies meanwhile reported programme costs as a participation barrier (Allen et al., 2011; Gibson, 2008).

### 3.6 Staff training

#### 3.6.1 Highly trained staff

Two studies used highly trained specialist nurses (Allen et al., 2011; Dean et al., 2014). One of these also employed a consultant with specialist expertise in hypertension for assistance (Dean et al., 2014), and the other used experienced nurse interventionalists rather than clinic nurses (Crowley et al., 2013). The studies' findings indicate that while highly trained staff may be more reliable, their availability to deliver prevention interventions in resource‐constrained areas may be lacking.

#### 3.6.2 Training for nurses

Included studies outlined several nurse training initiatives including training in motivational interviewing behaviour change techniques (Allen et al., 2011; Crowley et al., 2013; Fischer et al., 2012; Mertens et al., 2014; Murphy et al., 2015), health behaviour counselling (McKee et al., 2011) and cultural sensitivity (Crowley et al., 2013). Nurse training was often led and supported by senior and expert care staff on an ongoing basis (Shishani et al., 2019). Training benefits were largely unreported in the included studies. However, one study involving training at a rural community health centre was deemed effective in terms of both costs and improving nurses' knowledge / skill sets (Weiler & Tirrell, 2007).

### 3.7 Patient access difficulties

#### 3.7.1 Telephone/telemonitoring

Eleven studies used telephone technology initiatives. Telephone was used both as a primary and as an additional form of contact with patients (Crowley et al., 2013;Fischer et al., 2012; Jeong et al., 2018; Jordan et al., 2011); as part of follow‐up programmes (Dean et al., 2014; Piñeiro et al., 2020; Waller et al., 2016; Yang et al., 2016); and as a patient reminder tool between coaching sessions and screenings (McRobbie et al., 2016; Murphy et al., 2015). The studies indicate that telephone contact, as opposed to face‐to‐face contact, may decrease costs, and improve resource utilisation (Fischer et al., 2012;Jeong et al., 2018; Jordan et al., 2011). Telephone is also an accessible and familiar medium (Jeong et al., 2018; Jordan et al., 2011), and so it may facilitate more frequent contact between care providers and patients (Jeong et al., 2018; Kim et al., 2014), the overcoming of issues regarding travel‐ and / or time‐related care access restrictions (Jordan et al., 2011) and a greater sense of person‐centredness in sessions (Crowley et al., 2013; Jordan et al., 2011). Meanwhile, three studies used telemonitoring to combat limited resource access (Carter et al., 2011; Jeong et al., 2018; McKee et al., 2011), and this approach enabled frequent contact and closer relationships between patients and nurses (Carter et al., 2011).

#### 3.7.2 Alternative locations

Difficulties which low‐income patients can experience accessing care were also tackled using home‐based programmes and alternative settings. One study used CHW home visits (Gary et al., 2009) and four studies employed visiting nurses (Kim et al., 2014; Ko et al., 2011; McKee et al., 2011; Yang et al., 2016). Interventions were conducted at various alternate locations including: in community halls and hotels (Gibson, 2008); in religious settings (Brown et al., 2011; Monay et al., 2010); in schools (Brown et al., 2011); and on worksites (Murphy et al., 2015). The studies suggest that it is important that such locations are conveniently located and are accessible via public transportation (Murphy et al., 2015), as patients may feel at ease in familiar locations (Gibson, 2008), and leaders in community sites may be able to help care workers understand the socio‐cultural contexts of patients' communities (Murphy et al., 2015).

#### 3.7.3 Brief interventions

Included studies also showed that brief interventions can overcome barriers posed by time commitments. Numerous studies outlined brief intervention resources and strategies including clinical decision support tools (Bachhuber et al., 2017), brief motivational interviewing techniques (Mertens et al., 2014), brief advice interventions (Piñeiro et al., 2020) and short questionnaires (Waller et al., 2016).

### 3.8 Patient engagement

#### 3.8.1 Patient enrolment/interaction/retention problems

Numerous studies outlined patient engagement issues, their causes and strategies to overcome such problems (Brown et al., 2011; Dean et al., 2014; Froelicher et al., 2010; Gary et al., 2009; McRobbie et al., 2016; Mertens et al., 2014; Murphy et al., 2015). Difficulties regarding patient enrolment, interaction, and retention were common among the included studies, and competing everyday demands were cited as being the biggest cause of such issues (Froelicher et al., 2010). The included studies outline numerous initiatives and approaches that may counter problems like this including: the hiring of nurses who are familiar to (Dean et al., 2014) and liked by patients (Crowley et al., 2013); a high level of contact with nurses delivering interventions (Brown et al., 2011; Gary et al., 2009); flexible programme scheduling (e.g. reducing interventions to one session) (Gary et al., 2009); patient reminders via call, text, confirmation letter and / or email (McRobbie et al., 2016; Murphy et al., 2015), obtaining contact details for patients' friends / family (Gary et al., 2009; Mertens et al., 2014); and care provider‐ / patient‐led health goal setting (Murphy et al., 2015).

#### 3.8.2 Incentives

Incentives were also used in some studies to promote patient engagement with programmes. For instance, patients were offered contribution towards travel expenses (McRobbie et al., 2016), payment for attended sessions (Froelicher et al., 2010; McKee et al., 2011), gift cards (Mertens et al., 2014; Piñeiro et al., 2020) and opportunities to win prizes (Froelicher et al., 2010; Shishani et al., 2019). Several studies also outlined difficulties in retaining and recruiting nurses due to high demand for nurses in underserved, low‐income settings (Gary et al., 2009; Ko et al., 2011; Mertens et al., 2014). Staff incentives to address issues like this were not frequently discussed, but one study did note a smoking cessation intervention providing incentives for staff to distribute “quit kits” encouraging smoking cessation (Shishani et al., 2019).

#### 3.8.3 Family/friends support

Some primary prevention interventions involved patients' family and friends to overcome patient engagement issues and often with positive results. Successful initiatives of this nature included: patient participation in social networking modules (Carter et al., 2011); group education sessions (Murphy et al., 2015); patient grouping according to location to create feelings of neighbourhood support (Brown et al., 2011); and nurse‐led conversation regarding the accomplishments of familiar nearby participants to motivate patients (Yang et al., 2016). However, it is also worth mentioning that involving family and friend supports may be difficult (Weiler & Tirrell, 2007), although night / weekend programmes and explicit invitations for family / friends may address problems in this regard (Weiler & Tirrell, 2007). It is also worth noting that in some circumstances, family and friend involvement may impede intervention success. For example, family / friends may discourage patients from achieving intervention goals (e.g. smoking cessation) (Derksen et al., 2019).

### 3.9 Barriers faced by nurses

There was a lack of research regarding intervention barriers and facilitators. In studies which did examine this, barriers nurses faced included difficulty operating within existing GP practice hierarchies (Jansen et al., 2007), resource limitations (Ko et al., 2011), unmotivated patients (Derksen et al., 2019; El Fakiri et al., 2008) and inadequate training (Derksen et al., 2019).

## 4 DISCUSSION

### 4.1 Key findings

This study aimed to advance knowledge regarding the value and nature of nurse‐led behaviour change interventions to prevent CVD in disadvantaged communities. The findings suggest that interventions of this kind can be effective in preventing CVD, but the considerable variety of intervention approaches and outcome measures used to date makes it difficult to ascertain this. Factors enabling the success of interventions included tailoring interventions to specific populations, combatting patient access difficulties using remote care technologies, promoting patient engagement and providing adequate training for nurses. Cost‐effectiveness and intervention barriers were also explored but few studies examined barriers in depth.

### 4.2 How the findings relate to other literature

Previous studies have determined that nurse‐led interventions in non‐socio‐economically disadvantaged communities are effective in reducing CVD risk (Koelewijn‐van Loon et al., 2009; Tiessen et al., 2012; Voogdt‐Pruis et al., 2010; Wood et al., 2008), but that behaviour change interventions for vulnerable populations are challenging (Walton‐Moss et al., 2014). Despite this, our results suggest that nurse‐led behaviour change interventions can be effective in preventing CVD in socio‐economically disadvantaged communities. Previous studies have also cited the large variety of intervention approaches used for socio‐economically disadvantaged groups to date as a barrier to determining their effectiveness (Van Hecke et al., 2017). This review's findings do not dispute this point, but like Tan et al. (2020), they do indicate that a wide variety of targeted intervention approaches are necessary to meet the unique care needs of specific patient groups (e.g. older people, ethnic minorities, patients with low literacy/education). Barriers and facilitators cited in this review's studies that echo those reported in previous literature largely concern the nature and quality of relationships between nurses, patients and patients' communities (Michálková et al., 2016; Tan et al., 2020; Westland et al., 2018), as well as the availability of nurse resources (e.g. time, IT, physical space, training, support from physician peers) (Berra, 2011; Westland et al., 2020). Lastly, this review's findings are comparatively limited in the sense that patients' opinions and experiences of interventions were not widely examined by the included studies. Previous research has shown that patient engagement is affected by various psychological factors including patients' perceived physical and emotional benefits of interventions, as well as their personal level of goal attainment (Westland et al., 2019), and this review did not focus on this aspect.

### 4.3 Methodological considerations

The scoping review methodology used in this study allowed for broad mapping of the literature around nurse‐led behaviour change interventions to prevent CVD in disadvantaged communities. Arksey and O′Malley's scoping review framework was also beneficial, as it ensured that the study's research question development, study selection and data interpretation processes were conducted using a rigorous approach. The application of MeSH terms, electronic literature databases, the PRISMA ScR, Braun and Clarke's “Thematic Analysis” approach, and Levac et al.’s guidance were also helpful with regard to ensuring high methodological standards. However, this review also had limitations which should be considered. For instance, despite efforts to conduct a comprehensive literature search, it is possible that not all relevant publications were identified by the search strategy used. Furthermore, as is tradition with scoping reviews, an assessment of study quality was not conducted as we preferred to focus on mapping the findings of all relevant literature, regardless of study quality. The term “disadvantaged communities” used in this review was also quite broad. While we tried to narrow this definition by focusing on low socio‐economic status using income as the primary indicator, it is worth noting that the included studies' samples often varied considerably in terms of key demographic characteristics such as ethnicity and geographical location.

### 4.4 Implications for research, practice and policy making

Intervention initiatives tailored to disadvantaged groups, and strategies to overcome barriers impeding intervention success, should continue to be implemented and evaluated within health systems. For instance, this review's findings indicate that disadvantaged populations are considerably heterogeneous, and so future implementation / evaluation of primary prevention interventions should be conducted with the unique care needs and socio‐cultural contexts of specific population sub‐groups in mind. More research investigating patient experiences and perspectives of interventions will be useful when it comes to identifying what these specific needs and contexts are. System‐level barriers preventing nurses from delivering high‐quality interventions, and strategies tackling these barriers, should also be examined more closely. This study's findings suggest that numerous initiatives including telemedicine supports, visiting nurses, appropriately timed interventions sessions, community‐based supports, training, funding and incentives may be helpful with regard to addressing such issues.

## 5 CONCLUSION

Nurse‐led behaviour change interventions for high CVD risk patients in disadvantaged communities show much promise. However, disadvantaged populations are considerably heterogeneous in nature and future interventions may benefit from adopting approaches that are tailored to specific population sub‐groups' unique care needs. More research is needed to evaluate the potential of such interventions, as well as barriers and facilitators to positive intervention outcomes.

## CONFLICT OF INTEREST

The authors declare that there is no conflict of interest.

## ETHICS APPROVAL AND CONSENT TO PARTICIPATE

Not applicable.

## Data Availability

Data sharing is not applicable to this article as no new data were created or analyzed in this study.
